# Thinking on your feet: potentially enhancing phylogenetic tree learning accessibility through a kinaesthetic approach

**DOI:** 10.1186/s12052-024-00215-y

**Published:** 2024-11-11

**Authors:** Telma G. Laurentino, Meike Scheller, Gillian Glover, Michael J. Proulx, Alexandra A. de Sousa

**Affiliations:** 1 Department of Environmental Science, Policy, and Management, University of California Berkeley, Berkeley, CA USA; 2 Department of Psychology, Durham University, Durham, UK; 3 Department of Psychology, University of Bath, Bath, UK; 4 Department of Psychology, REVEAL Research Centre, University of Bath, Bath, UK; 5 Department of Anatomy, Physiology and Pharmacology, Auburn University College of Veterinary Medicine, Auburn, AL USA

## Abstract

**Background:**

Phylogenetics is one of the main methodologies to understand cross-cutting principles of evolution, such as common ancestry and speciation. Phylogenetic trees, however, are reportedly challenging to teach and learn. Furthermore, phylogenetics teaching methods traditionally rely solely on visual information, creating inaccessibility for people with visual impairment. Sensory learning style models advocate for tailoring teaching to individual preferred sensory learning style. However, recent research suggests that optimal learning, independently of preferred learning style, depends on the types of transmitted information and learning tasks. The lack of empirically-supported education into the effectiveness of teaching phylogenetics through alternative sensory modalities potentially hinders learning. The aim of this study was to determine whether phylogenetic trees could be better understood if presented in kinaesthetic or multisensory teaching modalities.

**Results:**

Participants (*N* = 52) self-assessed personal learning style and were randomly assigned to: visual, kinaesthetic or multisensory learning conditions. Phylogeny reading performance was better for both kinaesthetic and multisensory teaching conditions, compared to the visual teaching condition. There was no main effect and no interaction effect of personal learning style.

**Conclusions:**

This study establishes a baseline for further research by suggesting that easy-to-implement kinaesthetic teaching modalities might support phylogenetic tree learning and reading. This has practical implications for evolution education and accessibility for students with visual impairment, underscoring the need to shift from vision-centric teaching paradigms towards evidence-based instructional strategies that accommodate sensory diversity.

**Supplementary Information:**

The online version contains supplementary material available at 10.1186/s12052-024-00215-y.

## Background

Evolution is a fundamental concept to understand ecological systems and the global impacts of abrupt environmental change across biodiversity and society. Phylogenetic trees are one of the main methodologies of evolutionary biology research, which illustrate and anchor the understanding of core evolution concepts, such as: relatedness across biodiversity, common ancestry, adaptive divergence and speciation. However, students of evolutionary biology (Baum and Offner 2008; Meir et al. 2007) and professionals (Baum et al. 2005) alike consider learning to read and work with phylogenetic trees particularly difficult.

Furthermore, learners often have preconceived misconceptions about evolution that bias their reading of phylogenetic trees (Halverson and Friedrichsen 2013). There have been many attempts to quantify what drives these misunderstandings, and vision-based design alternatives to phylogenetic tree diagrams have been researched to prevent misreading (Gregory 2008), but it may be that learning phylogenetics can be better facilitated through different sensory modalities. However, to date, there is no research empirically comparing the effectiveness of learning phylogenetics when teaching through visual methods versus different sensory modalities.

Teaching effectiveness and knowledge retention has been linked to instruction methods and students’ preferred ways to process and understand information (Lethaby and Harries 2016; Aslaksen and Lorås 2018). It has been theorised that students tend to differ in their cognitive, psychological, and physiological approaches to learning with teaching sensory modality potentially playing a big role in learning success (Coffield et al. 2004).

Despite its common use, the available evidence for *learning styles* is conflicting. Few studies (e.g. Constantinidou et al., 2002; Massa et al., 2006; Cook et al. 2009) have used an effective experimental design, across disciplines and school ages, to accurately determine whether learning is indeed facilitated when teaching and learning styles match. While both adults and children have expressed preference for a teaching style that matched their own perceived learning style (Pashler et al. 2009), for college students, tailoring instruction to students’ learning style preferences does not significantly improve learning outcomes (Rogowsky et al. 2015, 2020).

Without integrative empirical approaches, conclusions are hindered and the effectiveness of individual learning styles remains heavily debated (Pashler et al. 2009). Despite the lack of evidence, learning styles are still applied in classrooms where the un-tested use of different sensory modalities can hinder learning instead of facilitating it (Constantinidou and Baker 2002). For example, a classic and popular model of sensory-based learning applied in classrooms is the VAK model (Dunn and Dunn 1978; Helena and Sreenidhi 2017; Scott 2010), which encompasses three main learning modalities: Visual, Auditory, and Kinaesthetic. While verbal learning ability can be facilitated by teaching within the visual modality condition, adding auditory information can be counter-effective (Constantinidou and Baker 2002). Recruitment of kinaesthetics seems to support cognitive processes when learning new complex tasks (Geary 2008; Paas and Sweller 2012; Damsgaard et al. 2022; Mathias et al. 2022; Andrä et al. 2020), but high bodily engagement has been linked to learning gains and also the risk of cognitive overload (e.g., Ruiter et al. 2015).

If presenting information with an inappropriate sensory modality is detrimental, then it is essential to identify which sensory modalities work best for which type of information. This is supported by perceptual studies showing that some tasks are better performed, and memory is enhanced (Lodge et al. 2016), when presented in a specific sensory modality: the modality appropriateness hypothesis (Hall 2016). This argument poses that effective learning of information depends on the optimal sensory modality that the information itself is presented in. This is extremely relevant in an education system over-reliant on visual forms of communication (Shabiralyani et al. 2015).

In STEM (Science, Technology, Engineering and Mathematics), teachers most often use visual aids, such as diagrams, graphs, or pictures, to facilitate learning. Not only can this hinder learning effectiveness when visual representations are not the most appropriate for a particular learning task (Reiner and Willingham 2010), but it obstructs inclusive education (Gray 2005; Karshmer and Bledsoe 2002; McCarthy and Shevlin 2017), contributing to the many barriers felt by STEM students with visual impairments (Bell and Silverman 2018). Evolution education fits the norm of lacking of multisensory alternatives to learning. Multisensory phylogeny activities have been created that apply auditory (Boutin and de Vienne 2017; Laurentino et al. 2021), kinaesthetic, and tactile (Halverson 2010; McLaurin 2013; Laurentino et al. 2021) information. However, these are not comparative studies disentangling which sensory modalities effectively increase the understanding of phylogenetic relationships between species.

Here we describe an activity testing phylogeny understanding across three sensory modalities. Participants with no extensive academic knowledge of phylogenetic trees were randomly split into visual, kinaesthetic and multisensorial modes of exposition to a phylogenetic tree. After their experience, participants answered a VARK questionnaire and a quiz to evaluate their level of phylogeny understanding. To eliminate confounding effects of pre-conceptions of biological relatedness (Halverson and Friedrichsen 2013), the presented phylogeny infers relatedness between fictional characters treated as Operational Taxonomic Units (OTUs). We test whether sensory modality and personal learning styles affect phylogenetic tree understanding scores.

## Methods

### Participants

An a priori power analysis using the G*Power3 (Faul et al. 2007) was conducted to determine the required total sample size to accurately test the first hypothesis. This showed that a total sample size of 64 participants, with three independent groups and a large effect size of *d* = 0.4 (Cohen 1988), was required to achieve a power of 0.80 with an alpha of 0.05. Our sampling size comprises 81.2% of the one suggested by the power analysis due to limited volunteer turnout and restricting research logistics. Participants were recruited from the University of Bath using a convenience sample resulting in 52 volunteers (35 Females, 17 Males; age range = 18–21). All participants self-reported to have normal or corrected to normal sensory ability. All participants self-reported no extensive academic knowledge of phylogenetic trees, nor extensive previous experience reading phylogenetic trees.

Three randomised learning groups were formed with: 18 participants taking part in the visual condition (9 F; 9 M), 17 in the multisensory condition (11 F; 6 M), and 17 in the kinaesthetic condition (15 F, 2 M). The global participant sample was not balanced regarding sex or post-experiment assessed learning styles (Supplement Figure S1). We chose to randomize group design to avoid experimental bias (Pashler et al. 2009). This randomization method resulted in the kinaesthetic group being the one with highest sex-inbalance (15 F, 2 M) and absence of auditory learners (Supplement Figure S1), which were overall rare in our participant body (7,7%) and are generally less common than other learner styles (Zhang 2011).

While this is not optimal, we found no evidence of the influence of sex in tree understanding score (Mann-Whittney U-test, W = 348, p-value = 0.2847), nor evidence for sex-bias on phylogenetic learning/interpretation in the current literature. We also found no evidence for dependence between sex and VARK learning type in our data (Fisher test, p-value = 0.347), which aligns with current literature with robust sample sizes (Urval et al. 2014; Dobson 2010). Thus, the experiment was run with randomization of participant’s demographics (Supplement Figure S1) and preferred learning styles (assessed post-experiment) across three sensory condition groups of quasi-equal sample sizes (18 visual; 17 multisensory; 17 kinaesthetic).

### Experimental Design and Procedure

Our experimental design follows suggestions within the field of learning styles (Pashler et al. 2009). All procedures were ethically approved by the Psychology Department Ethics Committee at the University of Bath.

**Fig. 1:**
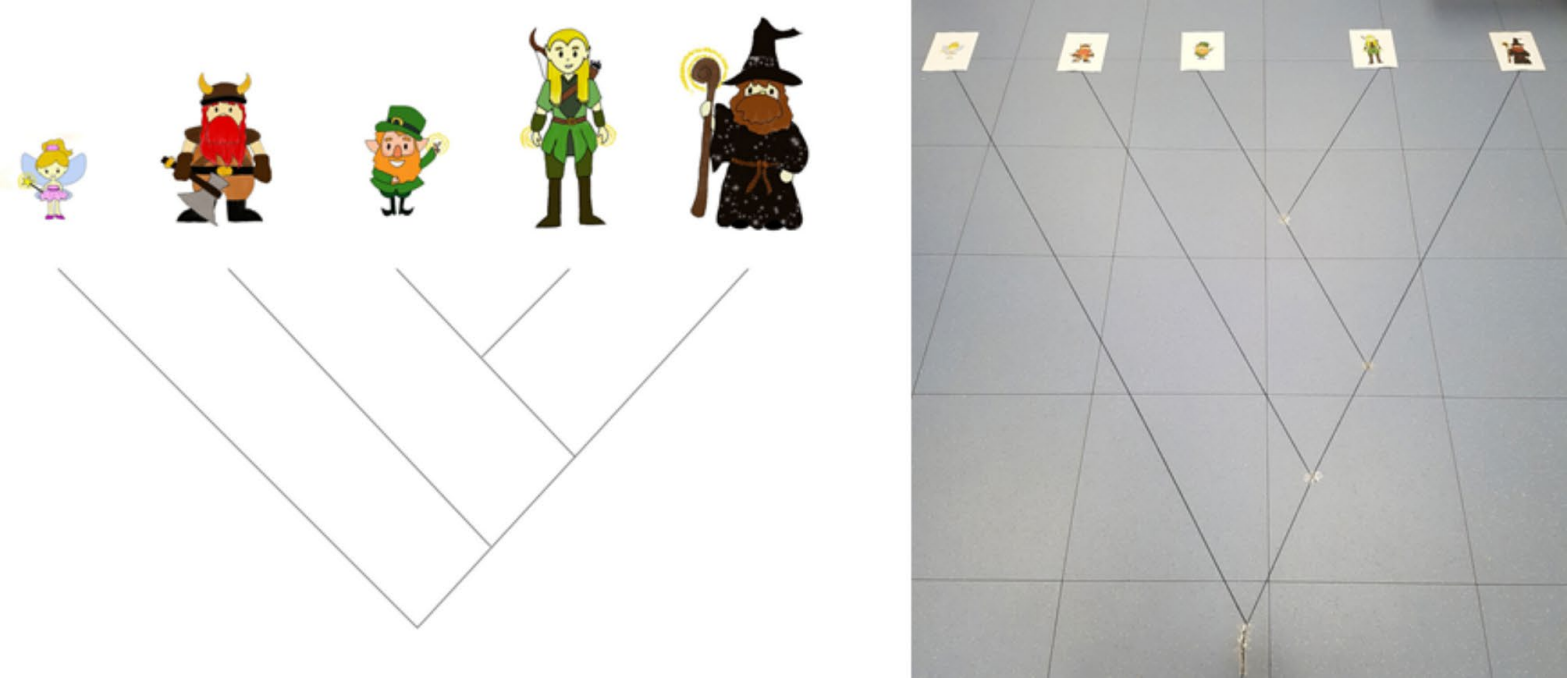
Experimental phylogenetic tree. **Left** panel shows the phylogenetic tree printed and provided in the visual condition; **Right** panel is a photo of the experimental kinaesthetic and multisensory floor set up. Alt text descriptions available in supplementary material 3

Five different Operational Taxonomic Units (OTUs) were placed on a phylogenetic tree, with lines representing the evolutionary relationship between them (Fig. 1). These OTUs consisted of five fantasy creatures designed by the experimenter, allowing the relation between OTUs to be arbitrary. That is, biological evolutionary relatedness could not be inferred by participant’s prior knowledge (Novick and Catley 2013; Halverson et al. 2011).

Participants were randomly assigned to one of three conditions. If in the visual condition, the participant remained seated and was given the phylogenetic tree print out (Fig. 1, left). If in the multisensory condition, the participant was guided over to the floor-laid out phylogenetic tree and asked to stand at the tree root (Fig. 1, right). If in the kinaesthetic (isolated egocentric spatial movement) condition, the participant was guided by the researcher through the floor-laid out tree, while consensually blindfolded to remove access to visual information.

Once a participant was exposed to the phylogeny, they were read a standardised script of phylogenetic basics (see the script in Supplement 2: Tree teaching and assessment) by the researcher. This included descriptions of the root as the oldest common ancestor to all represented OTUs (referred to as species in the standardised script; Supplement 2); branching as divergence caused by genetic or environmental change; and relatedness as shared common ancestry. It was made clear to participants that independently of the arbitrary nature of the OTUs represented, they are considered more related if they have a more recent shared ancestor which can be traced back to the phylogeny nodes. As the standardised script was read aloud to the participant, there was variation depending on the condition: to either look along the branches (visual condition), walk along the branches (multisensory condition) or follow along with the researcher guiding them along the branches (kinaesthetic condition).

**Fig. 2:**
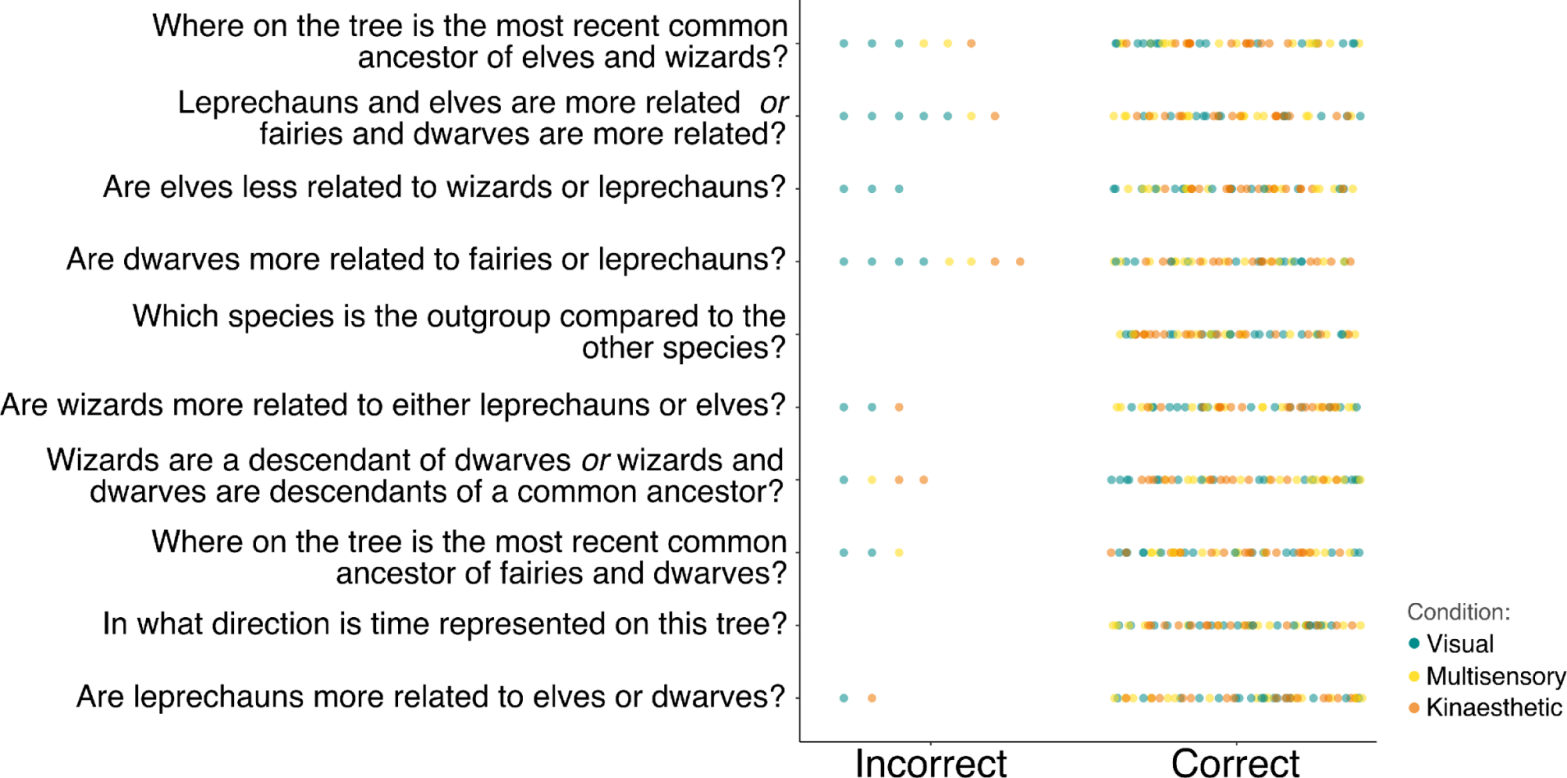
Scatter plot of the individual answers of the tree understanding assessment questionnaire. Each point marks a participant’s answer as correct or incorrect and is coloured based on the learning condition they were randomly assigned to (blue for visual, yellow for multisensory and orange for kinaesthetic). Alt text descriptions available in supplementary material 3

Phylogenetic tree understanding score per participant was measured through a questionnaire given verbally by the researcher, still in the presence of the phylogeny stimuli. Based on the assessment used by Baum and colleagues (Baum et al. 2005), the questions include assessment of time directionality, evolutionary relatedness, outgroup identification, patterns of descent, etc. (Fig. 2; Supplement 2: Tree teaching and assessment). Within the multisensory condition, participants were free to move anywhere along the tree branches at this stage, and in the kinaesthetic condition, participants were free to ask the researcher to guide them anywhere along the branches. Answers were recorded by the researcher. Higher scores indicate better tree understanding.

To assess the impact of learning style on tree-understanding, participants filled out the VARK learning style questionnaire (version 8.01; Fleming 2008) (Supplement 2: VARK assessment). The results of this questionnaire were calculated by the researcher and communicated to the participants together with their tree understanding score. Participants were then described the two other alternative experimental conditions and asked which one they would have preferred to learn with (“Preference” column in supplementary data file 4). This qualitative question served to compare our results with previous findings suggesting that students might prefer lessons taught in their own learning style.

### Statistical analysis

A two-way analysis of variance (ANOVA) was planned to determine the main effects of sensory teaching modality and personal learning style on tree-understanding scores, as well as the interaction effects of these two variables. No a priori tests were planned due to the non-directional nature of the first hypothesis (influence of sensory mode on tree understanding), with the second hypothesis predicting there would be no effect of learning styles on understanding.

**Fig. 3:**
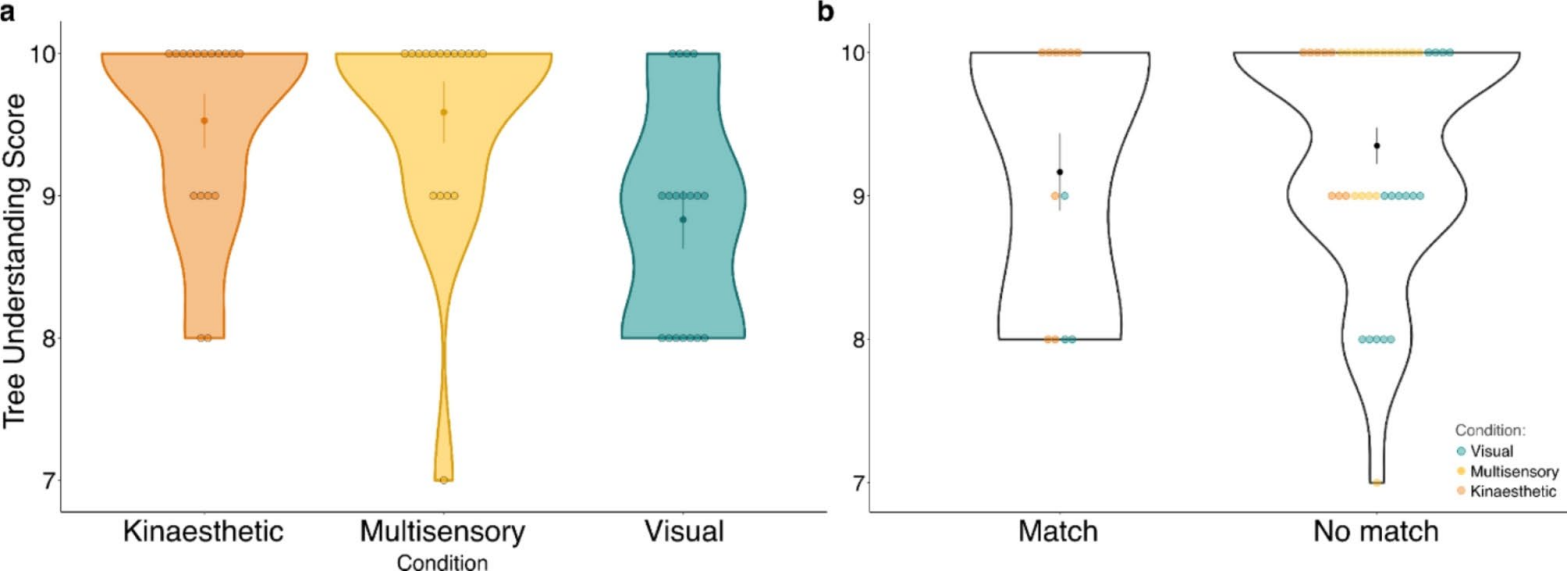
Violin plots with means and standard error (SEM) showing average tree understanding scores and individual data spread across sensory modalities. **a** plot shows tree understanding scores for different sensory learning conditions. **b** shows tree understanding scores for individuals whose sensory learning style assessed through VARK questionnaire was matched or not with the allocated learning condition. Each dot represents one participant and is colored by experimental condition. Alt text descriptions available in supplementary material 3

Averages were calculated for each participant’s tree understanding score (Fig. 3a), and the modal response was taken from their VARK assessment scores to assign a learning style.

Whether tree understanding differed between the three conditions using visual, kinaesthetic, or both sensory modalities was assessed using a non-parametric Kruskal-Wallice test for independent samples. A Wilcoxon rank-sum test was conducted to determine if tree understanding score was influenced by the alignment between the preferred learning style, assessed through the VARK questionnaire, and experimental condition (Fig. 3b).

Statistical analyses were conducted in R 4.2.2.

## Results

The majority of participants performed very well in the tree understanding questionnaire (Fig. 2). The average tree understanding score was 9.3 out of 10 (median = 10), with the maximum number of incorrect answers given by a single participant being 3 out of 10, which happened in only 1 case (Fig. 3). This high success rate is not surprising given that the participants were all brought to a basic understanding of the phylogeny through the scripted debrief (Supplement 2: Tree teaching and assessment) and answer the quiz directly after. Thus, the experiment evaluates capacity to retain and understand the given phylogenetic information and further interpret it across the sensory experimental conditions.

Majority of incorrect answers tended to occur within the visual condition (Fig. 2) where the participant sits in a chair observing the printed phylogeny. While no one erred questions on tree time directionality and outgroup species (Q5 and Q9, Fig. 2), the most incorrectly answered question asked if dwarves are more related to fairies or leprechauns (8 wrong answers), which illustrates a paradoxical difficulty in understanding time directionality in relation to internal node position.

This pattern was seen again when almost all participants (only 2 wrong answers) understood the greater proximity between sister branches of Leprechauns and Elves (Q2, Fig. 2),with understanding decreasing when needing to read more internal nodes on questions 2 (7 wrong answers) and 1 (6 wrong answers). Despite time direction being easily inferred from root to crown, it becomes more challenging to infer common ancestry relationships between tree branches as indicated by the patterning of the nodes. This difficulty is heightened for people learning in the exclusively visual condition (Figs. 2 and 3a).

We observed a main effect of sensory teaching modality (*Χ*(2,40) = 10.541, *p* = .005, *η*_*p*_^*2*^ = 0.174) on tree understanding scores (Fig. 3a). Bonferroni-corrected post-hoc comparisons indicated that the mean tree understanding score for the visual condition was lower than both the kinaesthetic (p_adj_ = 0.035) and multisensory conditions (p_adj_ = 0.015). Thus, tree understanding and interpretation increased with kinaesthetic information. Furthermore, the mean score for the multisensory and kinaesthetic conditions did not differ (p_adj_ = 0.999) indicating that phylogenetic tree understanding can be facilitated by kinaesthetic teaching modalities employing egocentric spatial movement.

We detected no main effects of VARK-assessed preferred learning style matching on tree understanding (*Χ*(1,40) = 0.054, *p* = .815; Fig. 3b). Thus, we find no evidence for the impact of personal learning style on tree understanding, as well as no interaction effect between personal learning style and teaching modality.

After learning the phylogeny in their assigned conditions and being told the results of their tree understanding quizz, participants were asked, if given a chance, which condition they would have preferred to learn in. That post-experience self-assessment revealed that participants only chose between the multisensory (94.2%) or kinaesthetic (5.8%) learning conditions, with a clear preference for multisensory conditions.

No one showed preference for the exclusively visual condition, despite the VARK questionnaire diagnosing 25% of participants as visual learners. The most frequent category of VARK personal learning style was Kinaesthetic (38.5%), followed by Reading/Writing (28.8%) and Visual (25%), with Auditory being the rarest category (7.7%).

## Discussion

Phylogenetic trees are a key crosscutting component to understand basic concepts of evolution. However, interpreting these diagrams is reportedly challenging to teach and learn through classical visual approaches (Baum and Offner 2008; Baum et al. 2005; Gregory 2008; McLaurin 2013; Meir 2007).

The higher tree understanding scores observed both in the kinaesthetic and multisensory conditions compared to the visual condition indicates that visual input had little additional effect on phylogeny understanding. This suggests that phylogenetic tree-thinking may require kinaesthetic thinking for comprehension. Many students struggle with the mental rotational aspects of correctly interpreting the relationships between species linked by ancestry nodes (Baum et al. 2005; Gregory 2008; McLaurin 2013). Indeed, mental rotation specifically takes place in the posterior parietal cortex in the brain; an area also linked to kinaesthetic learning (Seepanomwan et al. 2015; Zhang 2011). Additionally, research involving the congenitally blind tends to show that mental rotation does not require visual input (Marmor and Zaback 1976; Rovira et al. 2011). This may explain our results of higher effectiveness of both modalities including kinaesthetic information.

The lack of influence of VARK-assessed learning style preference on tree understanding score adds to growing empirical literature supporting the modality appropriateness hypothesis (Hall 2016): Learning style models should be applied depending on the nature of the task, rather than the personal learning styles of students. Thus, the over-reliance on visual models for evolution education might obstruct learning not only for students with visual disability, but all others.

It is important to acknowledge that this study has a limited participant sample size (*N* = 52) and thus, limited statistical power. The patterns that emerged suggest that kinaesthetic teaching may increase understanding and learning of phylogenetic trees, independently of student’s vision ability (here tested solely through blindfolding) or individual learning style preferences, but this hypothesis requires further testing. Namely, including participants within the blindness spectrum to determine whether similar learning effects occur, since people with visual impairment tend show differences in egocentric processing, particularly the adventitiously blind (Pasqualotto and Proulx 2012; Ruggiero et al. 2012).

Future studies should also consider the usage of completely artificial OTUs. Despite succeeding in avoiding misinterpretations due to assumed biological relatedness between known species (Novick and Catley 2013; Halverson et al. 2011), the folklore and mythological OTUs applied in this study may cause relatedness biases related to the participant’s cultural histories and media consumption .

In this study, students were asked if they had experience in phylogenetics, to which they all responded negatively. They were brought to the same basal information level through the standardized script when they first encountered the tree (Supplementary information). It would be interesting to also measure tree-understanding scores by applying the same questionnaire before and after sensory conditions, and see which different aspects of phylogeny misreadings can be aided or hindered by multisensory of kinaesthetic teaching methods for students with and without vision impairment, across different levels of evolution academic expertise .

We here establish a baseline for research into multisensory teaching of phylogenetics that has much needed reason to expand.

## Conclusions

This study establishes a baseline of research suggesting that phylogenetic trees can be better understood if presented in kinaesthetic and multisensory contexts, rather than the classical vision-centric approach to phylogenetics teaching. Our study adds to the literature showing that multisensory teaching approaches are not only be essential to ensure access for sensory diversity, but are efficient (Mathias et al. 2022; Andrä et al. 2020) and preferred (Urval et al. 2014; Laurentino et al. 2021) methods of learners in general.

Evolution outreach projects with the blind community (Laurentino 2019) highlight the over-reliance of general scientific education on visual and auditory stimuli, while neglecting haptic and kinaesthetic information. This contributes greatly to education barriers felt by people with visual impairment and the consequent low representation (less than 11% of PhDs) of people with any disability in the academic community (National Science Foundation, 2021).

Following evidence-based practices will allow education to better support more diverse student communities using different methodologies to think on their feet.

## Electronic supplementary material

Below is the link to the electronic supplementary material.

Supplementary Material 1

Supplementary Material 2

Supplementary Material 3

Supplementary Material 4

## Data Availability

All anonymous participant raw data will be made public with the manuscript as supplementary files 4. All material necessary for replication of the study are made public with the manuscript as supplementary files.
