# Supplementary material for: Thinking on your feet: potentially enhancing phylogenetic tree learning accessibility through a kinaesthetic approach
Source: Evolution (N Y). 2024 Nov 11;17(1):19. doi: 10.1186/s12052-024-00215-y (PMC11554830; doi:10.1186/s12052-024-00215-y)
Supplement: Supplementary file 1 — Supplementary Material 1 [file 12052_2024_215_MOESM1_ESM.docx]

**
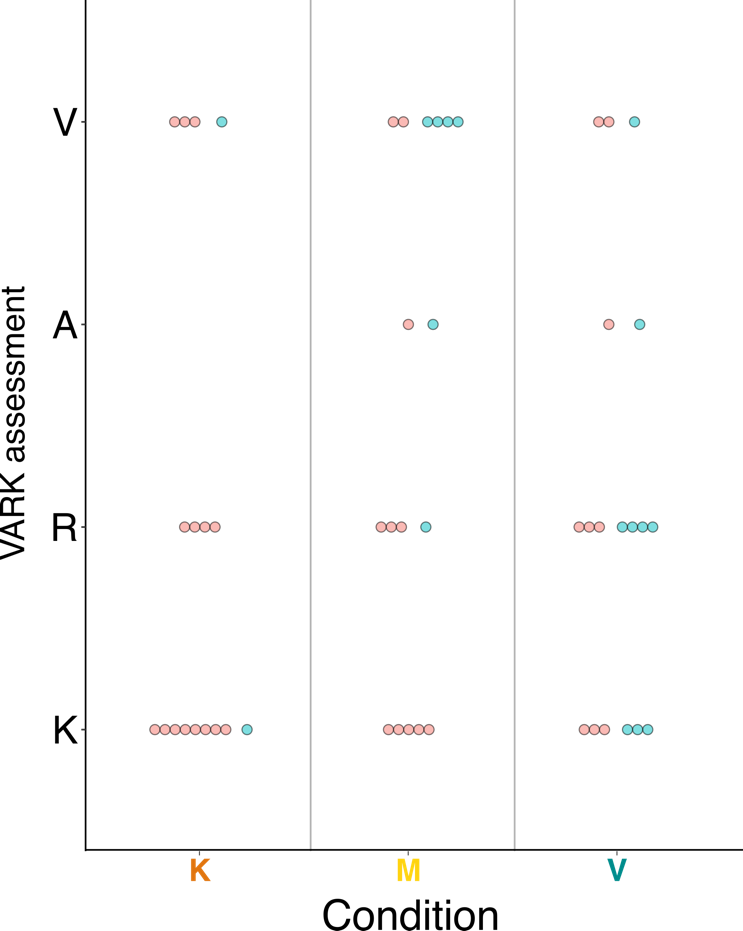
**

**Supplementary Figure S1: Distribution of participant demographics and VARK styles across experimental sensory conditions.** Each dot represents one participant, colored pink if female and blue if male. Auditory learners are the rarest (7.7%) and kinaesthetic are the most common (38.5%), with the remaining two types being intermediate: Read/Write (28.8%) and Visual (25%). Alt text descriptions available in supplementary material.
