# Supplementary material for: Thinking on your feet: potentially enhancing phylogenetic tree learning accessibility through a kinaesthetic approach
Source: Evolution (N Y). 2024 Nov 11;17(1):19. doi: 10.1186/s12052-024-00215-y (PMC11554830; doi:10.1186/s12052-024-00215-y)
Supplement: Supplementary file 2 — Supplementary Material 2 [file 12052_2024_215_MOESM2_ESM.docx]

**Supplement 2: Tree teaching and assessment**

**Trees Explained**

- A phylogenetic tree is a diagram that represents the evolutionary decent of an organism or set of organisms
- These trees are hypothetical, not factual, though they are informed by factual information that we have about different organisms, such as their physical features
- The tree demonstrates the relationship between species with branches, showing how they all descended from a common ancestor
- At the tips of the branches of a phylogenetic tree, you will find the organisms or groups of organisms that are of interest
- In this case, we are looking at Fairies, Dwarves, Leprechauns, Elves and Wizards. Don’t get too caught up in what the species are, this is an arbitrary tree designed for this experiment
- ***At the bottom of the page/where you are currently standing*** is the common ancestor for all of these organisms, which is also the furthest in the past
- Each split in the branches represents a divergence event, in which a group splits into two descendant groups
- There are several reasons that this may occur, such as changes in the environment or a genetic mutation
- This can lead to more obvious physical changes, or more subtle DNA variance
- Phylogenetic trees give us important information about the relatedness of two species
- Specific to phylogenetic trees, two species are considered more related if they have a more recent ancestor
- This ancestor can be identified by taking the two species and ***tracing back/walking back*** along the branches to find the internal node from which they have diverged from

**
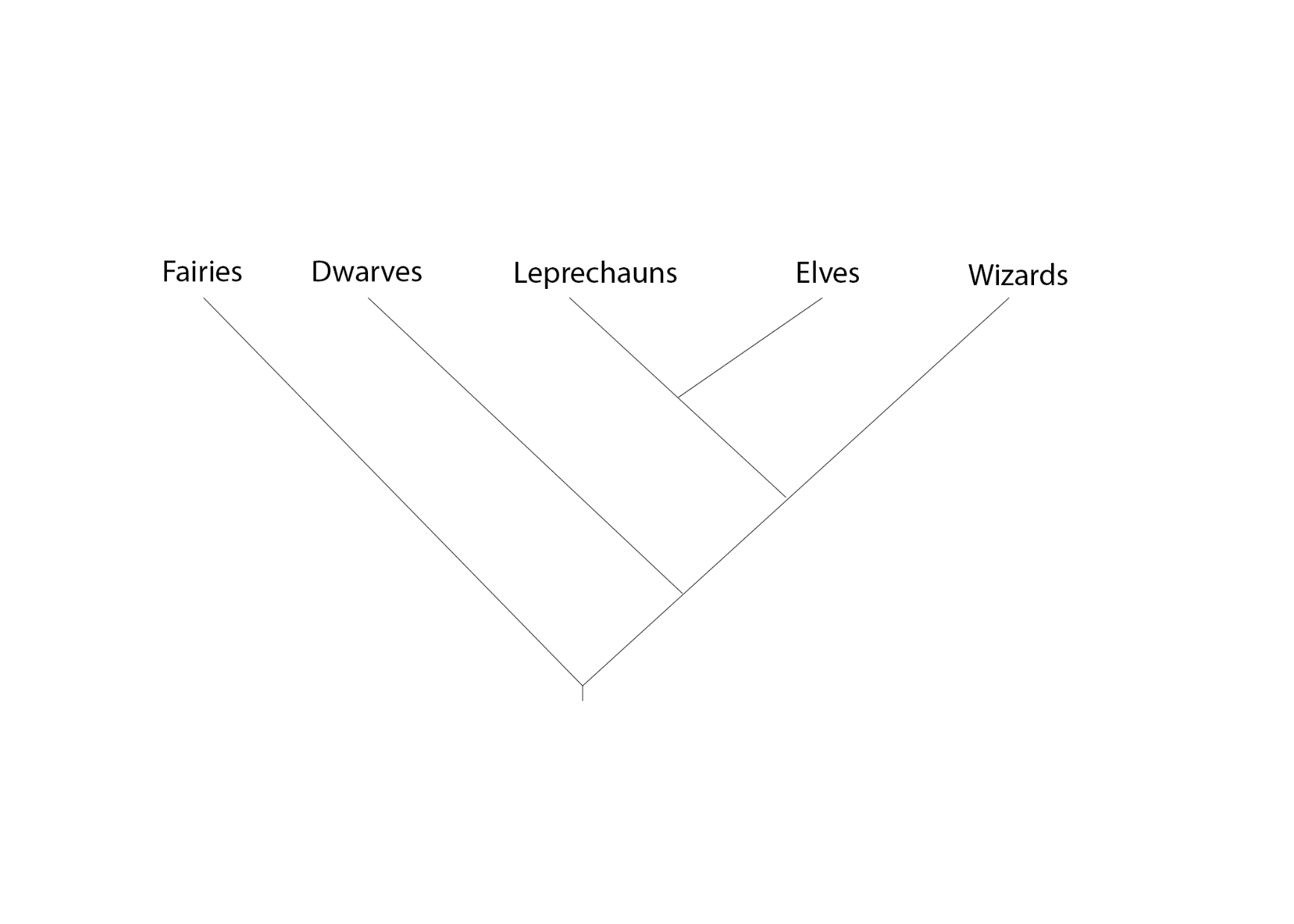
**

**Tree Understanding Assessment**

1. Are leprechauns more related to elves or dwarves?

Correct answer: Elf

1. In what direction is time represented on this tree?

Correct answer: Bottom to top, upwards

1. Where on the tree is the most recent common ancestor of fairies and dwarves?

Correct answer: First node, universal common ancestor

1. Which statement is correct: wizards are a descendant of dwarves // wizards and dwarves are both descendants of a common ancestor

Correct answer: Second statement

1. Are wizards more related to either leprechauns or elves?

Correct answer: No, they are equally related

1. Which species is the outgroup compared to the other species?

Correct answer: Fairies

1. Are dwarves more related to fairies or leprechauns?

Correct answer: Leprechauns

1. Are elves less related to wizards or leprechauns

Correct answer: Wizards

1. Which statement is correct: leprechauns and elves are more related // fairies and dwarves are more related

Correct answer: First statement

1. Where on the tree is the most recent common ancestor of elves and wizards?

Correct answer: The third node from the bottom

***Post-test question***

Would you have preferred or found it easier to learn about phylogenetic trees using the alternative methodology (visual, multisensory or kinaesthetic)?

**Supplement 2: VARK assessment**

**Learning Styles (VARK) Questionnaire**

This questionnaire is designed to give you an idea of what your learning style may be. However, do not consider this to be an absolute result; it’s just a helpful way of perhaps informing your future study. The learning styles included here are: Visual, Auditory, Reading/writing and Kinaesthetic.

In class, I prefer a teacher who uses:

Handouts, books, or readings R

Group discussion or guest speakers A

Demonstrations or practical sessions K

Diagrams, maps or graphs V

If I wanted to learn how to play a new game, I would:

Read the instructions R

Use the diagrams that explain the different moves or strategies V

Listen to others explaining the game and ask them questions A

Watch others play the game before joining in K

I am trying to make a gift for someone. I would:

Talk over ideas with a friend A

Make something I have made before K

Try to recreate something from a picture V

Find written instructions on how to make it R

If I were trying to assemble flat-pack furniture, I would:

Watch a video of someone assembling the furniture K

Use the written instructions that came with the furniture R

Ask advice from someone who had assembled it before A

Use the diagrams that show each stage of the assembly V

If I had a health issue, I would prefer that the doctor:

Showed me a diagram of what was wrong V

Described what was wrong A

Gave me something to read to explain what was wrong R

Used a plastic model to show me what was wrong K

I have a presentation to prepare for a class. I will:

Make diagrams and graphs to illustrate my points V

Use examples or stories to present my arguments K

Use written handouts for the audience R

Rehearse what I am going to say during the presentation A

I am receiving feedback from a test. I would prefer to have feedback:

From someone discussing it with me A

That used examples of what I had done K

As a written description of my results R

Using graphs to show what I achieved V

I want to find out more information about a new house. Before visiting it, I would prefer to:

View a video of the property K

Have a discussion with the owner A

Look at a plan showing the rooms and a map of the area V

Read a description of the rooms and features R
