# Supplementary material for: Thinking on your feet: potentially enhancing phylogenetic tree learning accessibility through a kinaesthetic approach
Source: Evolution (N Y). 2024 Nov 11;17(1):19. doi: 10.1186/s12052-024-00215-y (PMC11554830; doi:10.1186/s12052-024-00215-y)
Supplement: Supplementary file 3 — Supplementary Material 3 [file 12052_2024_215_MOESM3_ESM.docx]

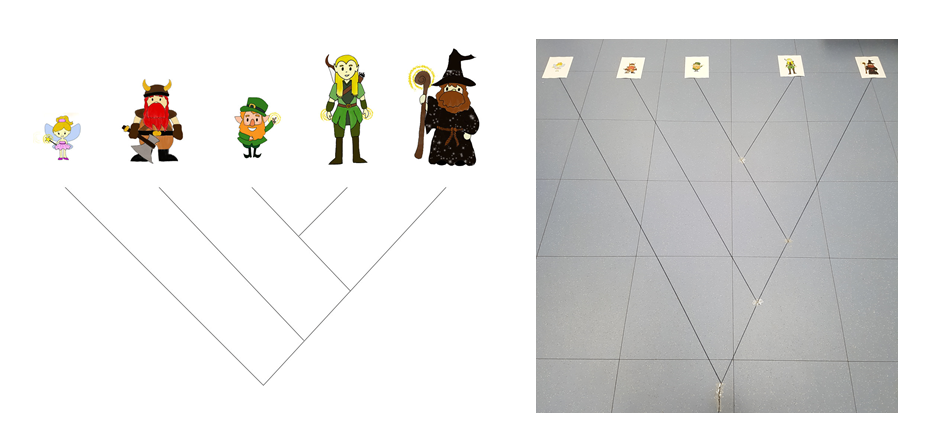


**Figure 1 legend: Experimental phylogenetic tree.** **Left** panel shows the phylogenetic tree printed and provided in the visual condition; **Right** panel is a photo of the experimental kinaesthetic and multisensory floor set up.

**Figure 1 Alt text: The figure depicts a phylogenetic tree with 5 branches with a fantasy species at its tip the final shape of the tree forms an inverted triangle. There is a main branch from the vertex of the triangle forming its right side, from which all other branches branch to the left. The first branch from the root, the outgroup, which forms the left side of the inverted triangle is the fairy. She is a small red-hair girl with big blue wings, pink dress, pink shoes, and a pink bow on her hair. She’s holding a magic wand with a star at its tip. Above it, along the right main branch, there is the branch of the dwarf. He is twice the height of the fairy. he has a horned helmet and a long red beard. His clothes look like war armour in tones of brown, he wears black boots and is holding an axe. Next, there is a branch that, towards the branch tip, bifurcates in two sister branches for leprechauns and elves. The leprechaun is slightly taller than the fairy (who is the smaller species), dressed in all green with a tall hat with a black ribbon and gold buckle. Under the hat his pointy ears are showing. He has a long red beard (lighter, more orange than the dwarf). He wears black boots and his left arm if lifted in greeting and a golden shine sparkles form his raised hand. The Elf is also all dressed in green, pants and a tunic, with knee-high brown boots. He is the tallest of all species and has long blond hair, pointy ears and he carries a bow and arrows. Both the elf’s hands show the same shimmering gold light as the leprechaun. Indicating they share their magic type. From the base of the branch containing the sister branches of the leprechaun and elf, branches the Wizard. All dressed in a long black tunic tied on the waste by a brown rope. He wears a big pointy black hat and has abundant and long brown beard and hair. In his right hand he holds a wooden staff about his height, with golden shimmer at the tip of the staff, indicating that his magic is accomplished through that object.**

**In sum, based on the fantasy phylogeny branching, the relationships between the species are: the fairy as the outgroup and most divergent species, followed by a common ancestor that split into dwarves and everyone else. Everyone else’s common ancestor then split into leprechauns and elves (sister species) and wizards. Thus, the most closely related species depicted are the leprechauns and elves as they share the most recent common ancestor.**

**The exact same pattern and figures are also depicted as a photo of the phylogeny reproduced on the floor with string. Spanning around 2 meters from root to branch tips. This is the floor phylogeny used to guide blind-folded participants in the kinaesthetic condition.**

**
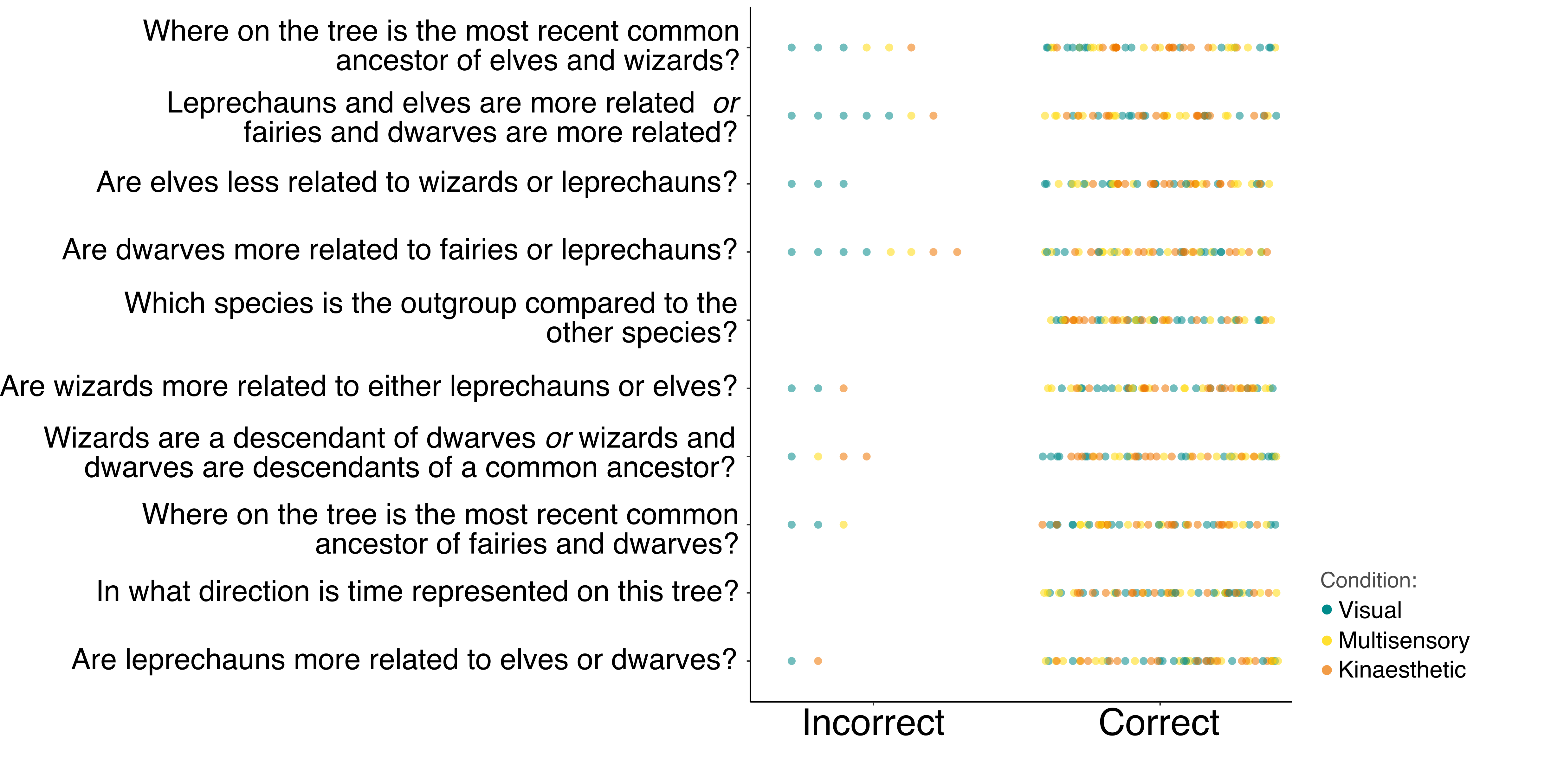
**

**Figure 2 legend: Scatter plot of the individual answers of the tree understanding assessment questionnaire.** Each point marks a participant’s answer as correct or incorrect and is coloured based on the learning condition they were randomly assigned to (blue for visual, yellow for multisensory and orange for kinaesthetic).

**Figure 2 Alt text: The plot depicted is a scatter plot organized in two columns (Incorrect and Correct) and 10 rows (one row per question asked to assess the participants’ tree understanding). Each row in the plot is populated by dots representing each participant’s answer, color-coded by the their randomly attributed learning condition (visual, multisensory and kinaesthetic). The first clear pattern of the graph is that there were few incorrect answers, with much more dots population each row of the correct column across questions. A second pattern is that the majority of the incorrect answers are concentrated in the following questions, by order: Are dwarves more related to fairies or leprechauns? (8 incorrect answers: 4 in the visual condition, 2 in the multisensory and 2 in the kinaesthetic); Leprechauns and elves are more related or daireies and dwarves are more related? (7 incorrect answers: 5 in the visual condition, 1 in the multisensory and 1 in the kinaesthetic); Where on the tree is the most recent common ancestor of elves and wizards? (6 incorrect answers: 3 in the visual condition, 2 in the multisensory and 1 in the kinaesthetic); There were no incorrect answers to the questions: In what direction is time represented on this tree?, and Which species is the outgroup compared to other species?; there are 4 incorrect answers in the question Wizards are a descendant of dwarves or wizards and**

**dwarves are descendants of a common ancestor?**

**, 1 from the visual condition, 1 from the multisensory conditions and 2 from the kinaesthetic condition; there are 3 incorrect answers in the question are elves less related to wizards or leprechauns, all from the visual condition; there are 3 incorrect answers in the question Are wizards more related to either leprechauns or elves?, 2 from the visual condition, one from the kinaesthetic condition; there are 3 incorrect answers in the question Where on the tree is the most recent common**

**ancestor of fairies and dwarves?, 2 from the visual condition, one from the multisensory condition.**


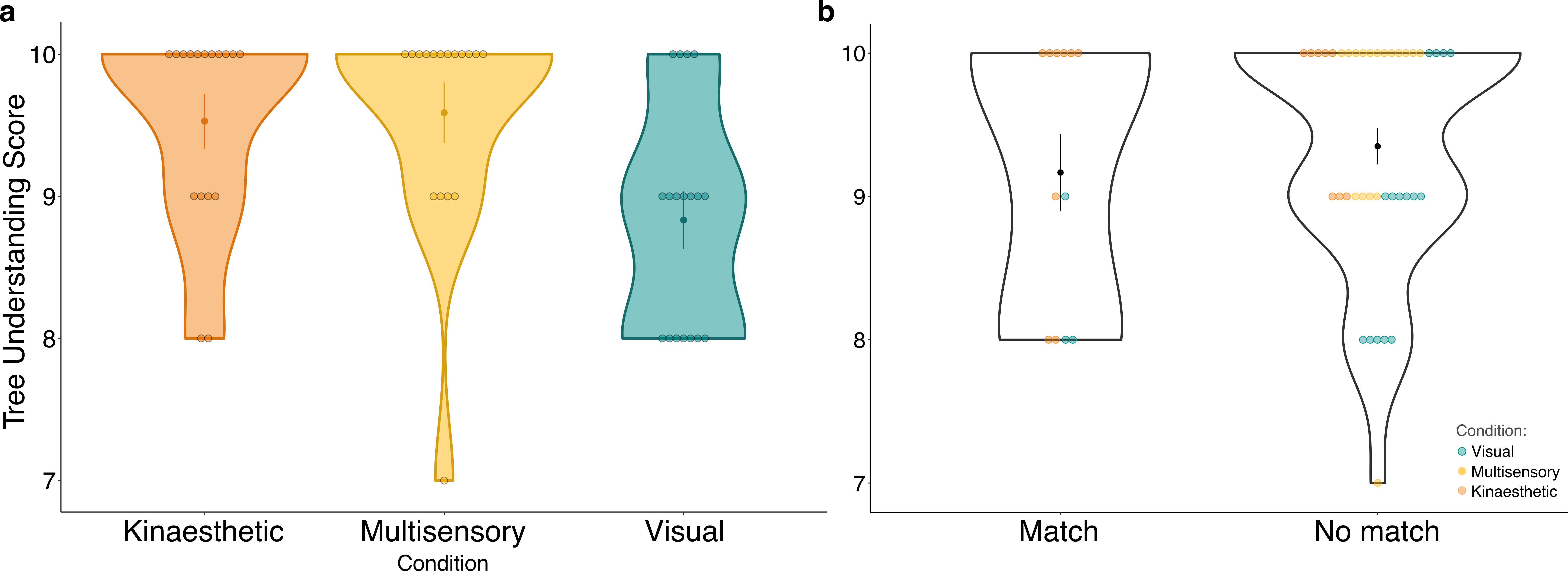


**Figure 3 legend**: **Violin plots with means and standart error (SEM) showing average tree understanding scores and individual data spread across sensory modalities**. **a** plot shows tree understanding scores for different sensory learning conditions. **b** shows tree understanding scores for individuals whose sensory learning style assessed through VARK questionnaire was matched or not with the allocated learning condition. Each dot represents one participant and is colored by experimental condition.

**Figure 3 Alt text: the figure consists of two panels with violin plots. The one to the left is organized in 3 columns on the xx axis concerning the sensory learning conditions attributed to participants: Visual, Multisensory and Kinaesthetic. The YY axis contains three understanding score, which is calculated as the number of correct answers to the 10 phylogeny-reading questions described in Figure 2. The main pattern we observe are higher averages and wider violin plots towards higher values of tree understanding for the kinaesthetic and multisensory conditions, by comparison to the visual condition. The majority of dots representing individual participant scores are concentrated on 10 in kinaesthetic and multisensory conditions, while they are equally distributed between 9 and 8 in the visual condition. Making the visual violin more evenly distributed than the other two. The longest violin tail is found on the multisensory condition, caused by a single point in value 7, meaning that the maximum number of wrong answers given by an individual participant was 3.**

**The plot to the right follows the same yy axis coding of tree understanding score, but the YY axis now depicts 2 columns: match and not match, referring to the match between experimental condition and the participant’s VARK-assessed prefered learning style. The main pattern shown is an absence of differences in average between the two, showing no effect of VARK-assessed preference in tree understanding outcome. Both violin plots show similar distributions, with the most noticeable difference being that the matched condition shows an almost binary distribution of points between the tree understanding score of 8 (4 points) and 10 (6 points), with only two points at the value 9. The not matched condition shows the higher density of points at value 10 (21 points), followed by 9 (13 points), followed by 8 (5 points) and 7 (1 point).**

**The majority of participants were thus not matched in their VARK-assessment and experimental condition but this does not seem to have statistically relavant influence in**

**tree understanding score between sensory learning conditions.**

**
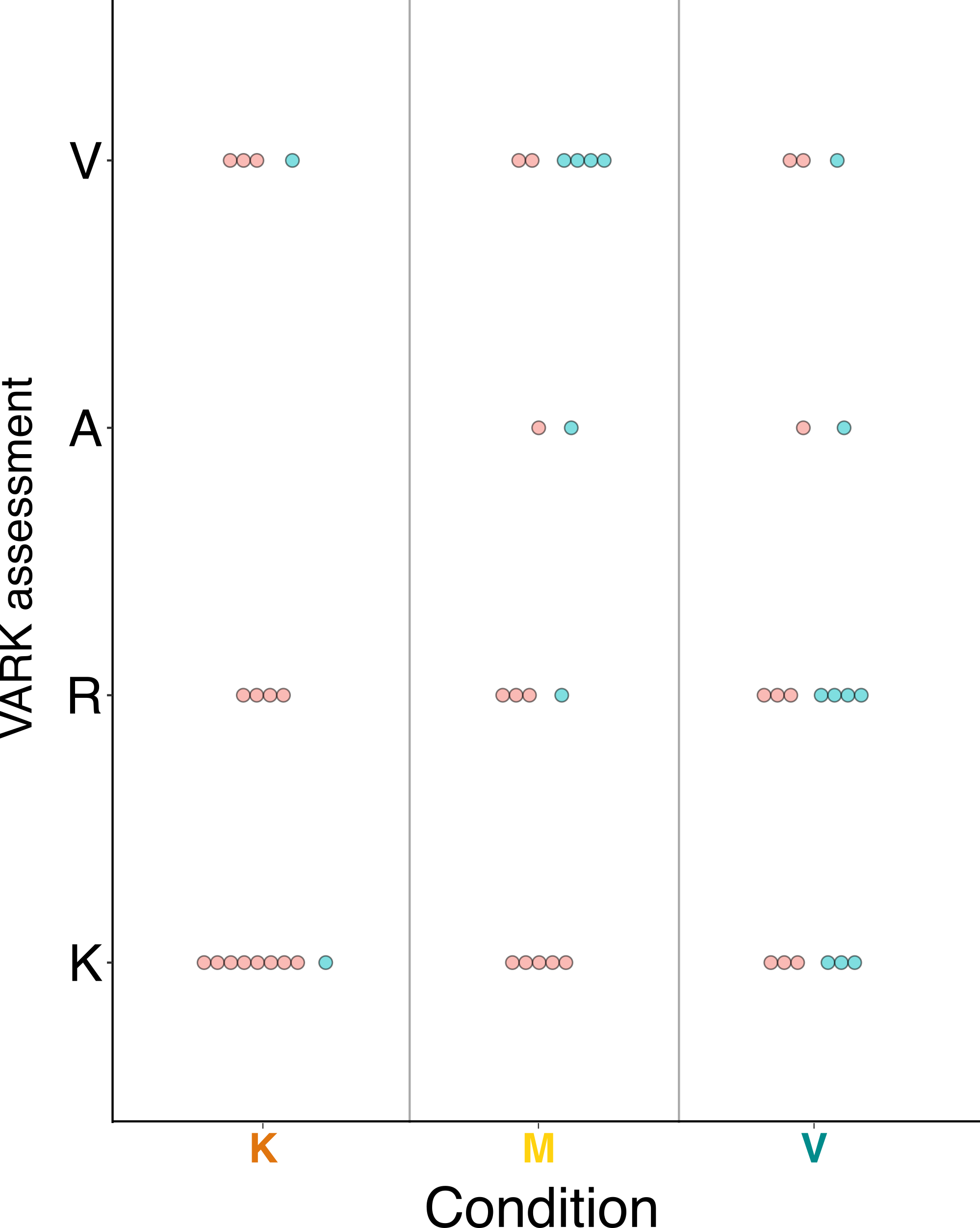
**

**Supplementary Figure S1 legend: Distribution of participant demographics and VARK styles across experimental sensory conditions.** Each dot represents one participant, colored pink if female and blue if male. Auditory learners are the rarest (7.7%) and kinaesthetic are the most common (38.5%), with the remaining two types being intermediate: Read/Write (28.8%) and Visual (25%).

**Supplementary Figure S1 Alt text: The plot depicted is a scatter plot organized in three columns (from left to right: Kinaesthetic, Multisensory and Visual). Four rows represent each of the categories assessed in the VARK questionnaire (Visual, Auditory, Read/Write and Kinaesthetic). Each row in the plot is populated by dots representing each participant color-coded by sex (female in pink to the right, males in blue to the left). The distribution of datapoints reveals that the group of participants allocated the Kinaesthetic condition was composed by a total of 15 Females and 2 Males. Of the Female’s VARK results, 3 ranked Visual, 4 ranked Read/Writing and 8 ranked Kinaesthetic. Of the Males’s VARK results, 1ranked Visual, and 1 ranked Kinaesthetic.**

**The group of participants allocated to the Multisensory condition was composed by was composed by a total of 11 Females and 6 Males. Of the Female’s VARK results, 2 ranked Visual, 1 ranked Auditory, 3 ranked Read/Writing and 5 ranked Kinaesthetic. Of the Males’s VARK results, 4 ranked Visual, 1 ranked Auditory, and 1 ranked Read/Writing.**

**The group of participants allocated to the Visual condition was composed by a total of 9 Females and 9 Males. Of the Female’s VARK results, 2 ranked Visual, 1 ranked Auditory, 4 ranked Read/Writing and 3 ranked Kinaesthetic. Of the Males’s VARK results, 1 ranked Visual, 1 ranked Auditory, 4 ranked Read/Writing and 3 ranked Kinaesthetic.**

**Overall, the plot depicts the rarity of preference for auditory learning styles and that the three experimental groups were balanced for total participant number with sex unbalance, especially in the kinaesthetic condition.**
